# Patient experiences with patient-reported outcome measures: an interview study of patients undergoing total hip– and knee arthroplasty

**DOI:** 10.1186/s41687-023-00561-3

**Published:** 2023-03-02

**Authors:** Laura Bjerg Mikkelsen, Niels Wedderkopp, Louise Schlosser Mose

**Affiliations:** 1 Department of Orthopedics, University Hospital of Southwest Jutland, 6700 Esbjerg, Denmark; 2 Department of Neurology, University Hospital of Southwest Jutland, 6700 Esbjerg, Denmark; 3 Department of Regional Health Research, University of Southern Denmark, 5000 Odense, Denmark

**Keywords:** Patient-reported outcome measures (PROMs), Hip and knee arthroplasty, Patient experience, Interview

## Abstract

**Background:**

Internationally, patient-reported outcome measures are increasingly applied in clinical settings to patients undergoing total knee arthroplasty (TKA) and total hip arthroplasty (THA). Current literature does not provide an understanding of the patient experience with these tools, as remarkably few studies are published investigating patient perspectives on completing PROMs. Thus, the aim of this study was to *investigate patient experiences, perspectives, and understanding with usage of PROMs for total hip and total knee arthroplasty in a Danish orthopedic clinic.*

**Methods:**

Patients who were scheduled for, or recently had, a THA or TKA for primary osteoarthritis were recruited to participate in individual interviews, which were audio-recorded and transcribed verbatim. The analysis was based on qualitative content analysis.

**Results:**

In total, 33 adult patients (18 female) were interviewed. Average age was 70.15 (range 52–86). The following themes were derived from the analysis: a) motivation and demotivation for completion, b) to complete a PROM questionnaire, c) environment for completion, and d) suggestions for use of PROMs.

**Conclusion:**

The majority of participants scheduled for TKA/THA were not fully aware of the purpose of completing PROMs. Motivation to do so arose from a desire to help others. Demotivation was affected by inabilities to use electronic technology. In terms of completing PROMs, participants expressed varied ease of use, and some perceived technical challenges. The participants expressed satisfaction with the flexibility of completing PROMs in outpatient clinics or at home; nevertheless, some did not manage completion on their own. Help was of great importance for completion, especially for participants with limited electronic capabilities.

## Introduction

A patient-reported outcome (PRO) is by The U.S. Department of Health and Human Services defined as “any report of the status of a patient’s health condition that comes directly from the patient, without interpretation of the patient’s response by a clinician or anyone else” [1]. Patient-reported outcomes measures (PROMs) are tools or instruments used to measure PROs [2], which ensure timely information from patients [3] and enable analysis of disease progression or regression as well as treatment effects [4]. PROMs are recognized as being positive for patients and care providers, as well as the health care system in general when used to evaluate the quality of health services [5, 6]. Recent systematic reviews identified following elements to be patient- or clinician-perceived benefits of PROMs; self-reflection, improved individualized approach, improved patient-clinician communication [7] promoted active patient involvement, enhanced the focus of consultations, improved quality of care, enabled standardized monitoring of patient outcomes, and enhanced the patient–clinician relationship [8].

The number of people undergoing elective hip and knee replacement has risen the past decade, and is in the Organization for Economic Cooperation and Development countries approaching a total of 2.5 million each year [9]. Following these procedures, PROMs have been considered important for evaluating the outcome, and routine implementation of PROMs into these programs has increasingly gained ground in many countries, including in Sweden since 2008, UK since 2009, and Canada since 2004 [10–12]. The international application of PROMs for these surgeries has been followed in Denmark. As there is currently no national, standardized follow-up of patients undergoing total hip arthroplasty (THA) and total knee arthroplasty (TKA), in 2016 the Danish Health and Medicines Authority started a national effort to develop and implement PROMs for these surgeries [13]. Whereas, the Danish clinical experience in capturing PROMs following TKA and THA is newly established. Annually, around 13,000 THAs and 9,500 TKAs are performed in Denmark [14, 15]. This study took place in one of the two hospitals that signed up to test the PROMs, where approximately 350 THAs and 350 TKAs are performed annually [16, 17]. A pilot test of the PROMs was run from February 2019 to December 2019 [13]. Although the interest in implementing PROMs has increased [3, 6], the existing literature has shown that doing so can be difficult [4, 6–8, 12]. In 2015, the response rate of patients in the UK undergoing hip and knee replacement was respectively 88.8 and 80.9 percent [18]. Also, statistics derived from the Danish pilot test clarified challenges in follow-up, as it was respectively 42 and 49 percent among THA and TKA patients at three months post-surgery [19]. Thus, it is unknown how a considerable number of patients manage post-surgery. This raises questions about mechanisms that affect completion of PROMs to understand why low response rates occurs in a Danish population. The literature on this aspect is limited, and a recent review identified only two studies concerning the use of PROMs as an integrated part of clinical practice regarding osteoarthritis [20]. Although PROMs have been developed with the intention of capturing patient-centric concepts, a gap exists in understanding the patient experiences with these tools, as remarkably few studies are published investigating patient perspectives on completing PROMs. A British study by Rowlands et all points out that participants were often interrupted while completing PROMs in the orthopedic department, which affected their ability to concentrate and raised anxiety about not completing the survey to the best of their ability. Furthermore, the participants had limited understanding of the purpose of completing PROMs [21]. Patients’ first-hand experiences with PROMs could provide unique knowledge concerning challenges as well as favorable experiences associated with the application of PROMs into clinical practice. Therefore, the aim of this study was to build upon the findings of Rowland et al. when *investigating patient experiences, perspectives, and understanding with usage of PROMs in total hip and total knee arthroplasty in a Danish orthopedic clinic.*

## Methods and materials

### Design

This study was conducted as a qualitative exploratory study based on semi-structured interviews. The design was guided by a hermeneutic inductive approach to analysis and interpret patients’ experiences, perspectives, and understanding with usage of PROMs in a content analysis.

We used the content analysis approach, as this is appropriate when existing research results for a phenomenon are limited [22, 23].

#### Participants and recruitment

To gain in-depth insight into patient-centered evaluation of orthopedic PROMs and obstacles to their completion, a maximum variation sampling strategy was used [24]. We aimed for maximum variation in gender, age, type of operation, and response status by contacting equally men and women in different age categories, with an equal number of participants operated for TKA and THA. Participants were eligible to participate in interviews if they were: > 18 years, Danish language skilled, diagnosed with either knee or hip arthritis using the international classification of diseases M16 or M17, and signed up for either TKA or THA. The recruitment took place from the orthopedic department’s two outpatient clinics. When the participants were signed up for surgery, the clinic staff introduced them to PROMs. Participants were able to complete PROMs using an available device in the outpatient clinics or by using their own electronic device. If the clinic staff were able to make time, they helped participants to either download the app or go to the website. Choosing to complete through the app required it to be downloaded, and to log in using a digital signature. If participants did not bring the digital signature or clinic staff were busy, participants received written take-home instructions for accessing PROMs. The pre-surgery PROMs expired 35 days after their appointment in the outpatient clinic. The possibility to complete three-month PROMs expired after 14 days. Participants were reminded to complete three-month PROMs in the app and in their personal secured electronically mailbox. The workflow for handling PROMs was the same in different outpatient clinics. The clinic staff identified eligible participants and offered them written and verbal information about the study. Participants who accepted to be contacted received a phone call from the researcher LBM, inviting them to an interview.

### Material

Individual face-to-face interviews were conducted to get insight about participants’ experience with reporting their data using a newly developed PROM scheme.

#### The PROM scheme

A national committee of 40 clinicians collaborated with The Danish Health and Medicines Authority to develop two PROM questionnaires; one for patients with hip arthrosis and one for patients with knee arthrosis [13]. In total the PROM scheme consisted of 33 items for THA patients and 36 items for TKA patients [13]. The schemes consisted of international validated PROMs: Oxford Hip/Knee Score, EQ-5D-5L, and Harris Hip Score. Also, Copenhagen Knee ROM Scale, a Danish national validated PROM [25], was included to examine movement of the knee in TKA patients. Elements from the Services, Pathways, Access, Research and Knowledge (SPARK)-study [26, 27] were included to examine pain-relieving medication. Also, elements from a Danish project called GLA:D were included to uncover labor market affiliation. GLA:D (Good Life with osteoArthritis in Denmark) is evidence-based education and supervised neuromuscular exercise delivered by certified physiotherapists nationwide targeting people suffering from osteoarthritis in hip or knee [28]. GLA:D has become widespread internationally with the intention to minimize pain through training[29]. Including the PROM scheme contained questions regarding assistive devices, training, weight loss, and personal goals [19]. It was decided that the PROMs were to be distributed using an application (app), named *Mit Sygehus*, and a website www.mit.rsyd.dk [30].

Participants were handed a written guidance that provided a description to navigate to the PROM questionnaire. First, they had to download the app, Mit Sygehus, or navigate to the website www.mit.rsyd.dk . Then participants had to select the suitable hospital and the treatment course they were attending. Afterwards participants had to log on using their personal digital signature by choosing “My account” and then “log on”. Dialogue boxes guided participants to sign for consent to allow the app to access data from their personal electronically journal. To navigate to the questionnaire the participants had to choose the suitable treatment course and then “questionnaire”. Most of the questionnaire were answered using Likert Scales, but some also contained pictures to illustrate e.g. degree of knee movement.

#### Data collection

From September 2019 to January 2020, LBM completed semi-structured individual interviews. Participants were interviewed once in relation to completion of pre- or three months post-surgery PROMs [13]. In order to investigate the research question, we decided to recruit participants into three groups, based on their response status:

1. Patients who completed the PROMs
2. Patients who wanted to complete the PROMs, but did not manage to
3. Patients who did not wish to complete the PROMs

Data from group one (G1) clarified general experiences with PROMs and possible challenges of completion. Data from group two (G2) clarified experiences with PROMs, and were especially focused on barriers for completion. Data from group three (G3) clarified reasons for refusing participation. Prior to interviews, the researchers LBM and LSM developed semi-structured interview guides for each group, guided by Kvale and Brinkmann [31]. The interview guides were based on existing literature and the focus of the study. Interviews were initiated with an introductory question: “*Try to tell me what you were told about the electronic questionnaire*” [31]. Wording such as “*Please describe in more detail”* was used to gain elaboration of the participants’ experiences [31]. The interviews were audio recorded and were, on average, conducted 52 days after participants were introduced to PROMs at the outpatient clinics.

### Analysis

Recordings from the interviews were transcribed verbatim by LBM using a transcription guide developed with inspiration from Kvale and Brinkmann [31]. Identifying data were removed to secure anonymizing of participants. All transcripts were uploaded to NVivo (version 20) where the analysis was performed, guided by the qualitative content analysis described by Graneheim and Lundmann [22]. Table 1 illustrates the steps taken during analysis.

**Table 1:** The steps during analysis, inspired by Graneheim and Lundmann

All transcripts were read multiple times to obtain a sense of the whole
The text was divided into meaning units which were condensed
The process of abstraction involved labeling the condensed meaning units with codes
The codes were arranged into categories and subcategories, based on assessments of their similarities and differences
A process of reflection and discussion between LBM and LSM resulted in agreement about how to sort the codes into categories
Finally, the underlying meaning, that is, the latent content, of the categories was formulated into a theme

Throughout the process of analysis, a back-and-forth movement happened between the whole and parts of the text [22]. When a new category was created, it caused reassessment of previously coded data, followed by recoding where appropriate. Table 2 illustrates an example of the analytic process.

**Table 2:** Example of the analytic process

Meaning unit	Condensed meaning unit	Code
“Then I was told that it came on, that I should complete it on an app or whatever you call it. And then I said, I am simply not able to do that (…) I am insecure about electronics.” (I20)	Electronic inabilities limit completion and lower motivation to complete PROMs	Motivation and demotivation for completion

### Ethics

According to the Declaration of Helsinki, the participants were informed that they had the right to withdraw from the study at any time [32]. They were assured that their responses would remain confidential and that their identity would not be revealed in any phase of the study. Signed informed consent from all participants was obtained before interviewing [31, 32]. The Region of Southern Denmark granted the appropriate ethical approval (22/35848).

## Results

**Fig. 1:**
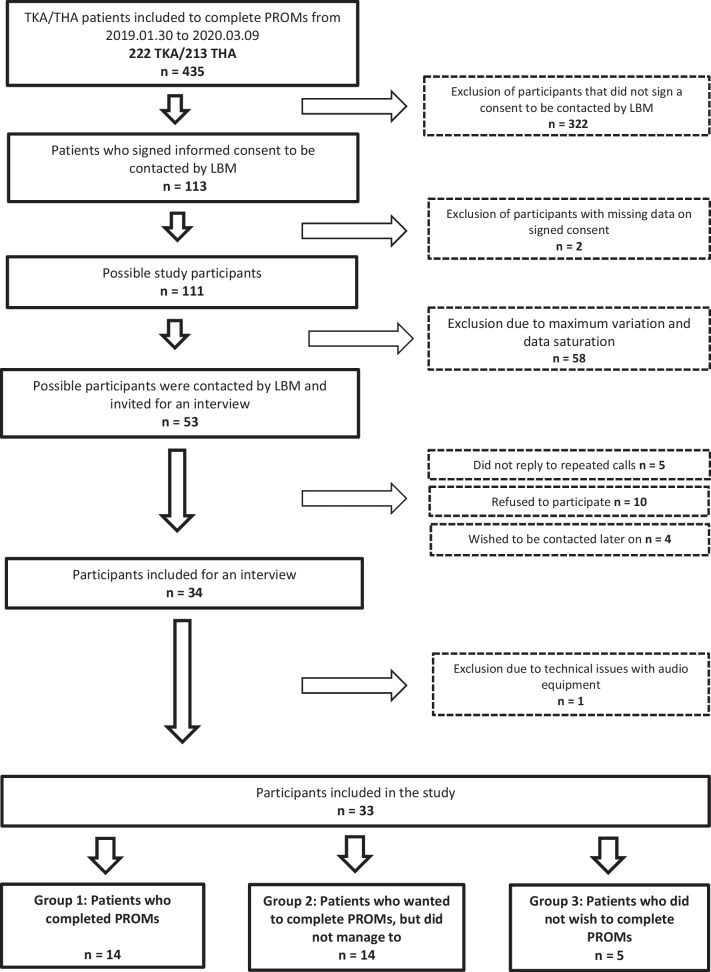
Flowchart

At the Orthopedic outpatient clinic a total of 435 THA and TKA patients received information about PROMs preoperatively from January 2019 to March 2020. Of those 268 (61 percent) completed PROMs. One hundred thirteen (26 percent) accepted to receive a phone call from LBM. Recruitment stopped when no new information arose from interviews [31], which resulted in a total of 33 participants. Figure 1 illustrates the process of inclusion.

Demographics of participants are listed in Table 3.

**Table 3:** Demographics of participants in the three groups

	Group 1: Patients who completed PROMs *n* = 14	Group 2: Patients who wanted to complete PROMs, but did not manage to *n* = 14	Group 3: Patients who did not wish to complete PROMs *n* = 5
Mean age	*66.2* (55–79)	70.8 (52–86)	79.4 (76–83)
Female	8	7	3
Type of operation (TKA/THA)	8/6	8/6	1/4

Participants had different preferences concerning place for conduction of interviews; 27 were interviewed at their homes, three at the hospital, and three at their workplace. In some cases, a participant’s spouse was present during interviews conducted at the participant’s home. Interviews lasted an average of 10 min. There was a great variation in the length of interviews between participants across groups, due to a big diversity in terms of how much they remembered about PROMs. In G1 some participants completed the PROMs with ease, and had not much to tell. While others experienced challenges during completion which caused longer interviews. Participants from G2 did not manage to complete PROMs, some did not manage to navigate to the questionnaire which caused short interviews, while others had more to tell about the challenges of completion. The shortest interviews were completed among participants from G3, as the interviews only revolved why they refused to complete the PROMs.

The structured analysis resulted in four major themes with subthemes, as presented in Table 4.

**Table 4:** Themes and subthemes

Themes	Subthemes
Motivation and demotivation for completion	Information perceived about PROMs Preparedness to receive information Electronic capabilities
To complete a PROM questionnaire	Ease of use Technical challenges
Environment for completion	Significant helpers Flexibility in completion
Suggestions for use of PROMs	User-friendliness

### Motivation and demotivation for completion

A central theme that emerged from the analysis was the participants’ motivation to complete PROMs. The motivation was influenced by the information perceived about PROMs, preparedness to receive information, and electronic capabilities. In most of the interviews, the participants had to be reminded of the content of the PROMs. Participants described how many activities happened on the day they were scheduled for surgery; for some, it could be difficult to remember the information they had received about PROMs.

Based on the interviews, it became clear that the information perceived about PROMs in the outpatient clinics varied greatly. Some participants remembered receiving information about PROMs, while others did not remember the clinical staff giving any information. The majority of participants in all three groups had a sparse understanding of the use of PROMs and only five participants understood that the PROM scheme was a tool used for follow-up. Most of the participants did not manage to explain the use of PROMs. Some thought that the reported data were used for science while others thought PROMs were a newly introduced examination in the outpatient clinics, as illustrated by I5: “*They [PROMs] are used in relation to research and in relation to ensuring other people getting well, it is not for my own sake”* (male, 67 years, G2). Some participants only remembered information being given about how to complete PROMs. Five participants in G2 did not remember receiving any information about PROMs. Hence, we must assume that the information did not seem important to the participants or that they were not able to accommodate all the given information.

Despite the sparse understanding of PROMs, all participants in G1 were motivated enough to complete it. In addition, some participants in G2 also expressed motivation to complete PROMs even though they did not succeed single-handedly. The expressed motivation seemed to occur from a willingness to help others; either scientists, clinic staff, or future patients, as illustrated by I14: *“I do not mind participating if it contributes to making you [health professionals] wiser or helps future patients”* (male, 66 years, G1). A few participants were concerned about what their data were used for, and expressed that they were worried if the reported data would influence their treatment, as illustrated by I15**:** *“If the answer was put in the first box [regarding pain], which is the mildest degree, then they might not operate on me. How can it be used against me? But I answered how I felt”* (female, 57 years, G1). This indicated that she worried about the postponement of surgery and considered rating pain higher than experienced to avoid postponement.

Regardless of the perception of PROMs, a total of 12 participants from G1 and G2 agreed that the timing of receiving information at the outpatient clinics was appropriate, as illustrated by I1: “*Actually, I think it is a well-chosen time to say, ‘By the way, do you want to participate?’ because you are already prepared to think along those lines”* (female, 61 years, G1)*.* Interviews conducted with participants from G1 pointed to a willingness to receive information from the clinic staff, and participants felt mentally capable of understanding this information. However, this is ambiguous, as the majority were not able to explain the use of PROMs. Many participants from G1 and G2 agreed that their motivation to fulfill PROMs had not been affected by the timing of information nor by the amount of information they received at the outpatient clinic. The amount of information, therefore, cannot be considered as a main cause for non-response in this study. However, it seems that the amount of different information could have made it difficult to remember it all.

Electronic capabilities was another subtheme, with a high impact on the participants’ motivation to complete PROMs. From interviews, we found that electronic capabilities varied from those who, according to themselves, could not use electronics, to those who perceived themselves to be experienced using electronic devices. In G1, slightly more than half of the participants reported being experienced in the use of electronic devices, while the remaining indicated they were inexperienced. As all participants in G1 completed the PROMs, their electronic skills did not seem to be an obstacle or affect their motivation to complete. In great contrast, participants in G3 were demotivated to complete PROMs due to their own electronic inabilities, as illustrated by I20: *“Then I was told that it came on, that I should complete it on an app or whatever you call it. And then I said, ‘I am simply not able to do that (…) I am insecure about all this electronics’” (female, 77 years, G3)*. All participants in G3 expressed that they could not manage completing PROMs due to insecurity about electronics. After completing four interviews, not much new data occurred. They all recommended administration of either paper based PROMs or gathering data through conversation with a health professional to enable completion as illustrated by I9: “*If you sent me one [a PROM questionnaire] and I just had to fill it out, it was no problem at all (…) in paper form right*”. By a few participants across all groups, it was problematized that they had to learn to use technical devices in their senior years, which was not easy. The number of self-reported inexperienced users of electronic devices was slightly higher among participants in G2 compared to G1. Overall, the participants’ inexperience did not seem to affect their motivation to complete PROMs; nonetheless, the completion was challenging in other ways.

### To complete a PROM questionnaire

Several elements affected the process of completing PROMs, especially the ease of use, and the experience of technical challenges.

Most participants from G1 completed the PROMs with ease, as illustrated by I26: “*I received a take home guidance, that clearly guided me (…) I thought it was easy [to navigate to the questionnaire], as I was guided through the user interface”* (male, 68 years, G1)*.* Those who perceived themselves experienced using technical devices did not typically have difficulty using the app or website. In most cases, participants preferred to use the app when completing PROMs. The majority of participants in G1 indicated that it was easy navigating to the questionnaire. In contrast, most participants in G2 experienced different technical issues that challenged completion of PROMs. The biggest challenge was navigating to the questionnaire. Furthermore, participants expressed technical challenges e.g., to log in, to stalled the app, and because pictures of knee movement were not presented in the app.

In relation to usefulness and readability, there were divergent opinions. The majority of participants from G1 found that the questions were easy to understand and expressed that the PROMs fully covered the state of their condition. However, other participants needed to read a few of the questions several times to fully understand how to answer as illustrated by I17: *“some of the answers were closely related (…) sometimes I needed to evaluated if answer number four or five best fitted [his condition]”*(male, 75 years, G1). This was especially the case for the questions related to severity of pain; these were stated as challenging to answer by almost half of the participants that completed PROMs, as illustrated by I16: “*It is not easy to tell, sometimes I feel no pain and other times the pain increases (…) and you are only allowed to put one cross*” (female, 69 years, G1). Difficulty occurred due to variation in pain during the day, the impact of painkillers, and the distinguishing of other comorbidities.

Regarding the length of the questionnaire, most participants felt it was appropriate, requiring a range between 10 to 20 min to complete. A few participants reported the pictures in PROMs to be useful, as it made it easier to select an appropriate answer.

### Environment for completion

Central elements that emerged under this theme during the analysis were significant helpers, and the flexibility in completion.

In both G1 and G2, many participants expressed that helpers to complete the PROMs were of great importance. Mainly, the clinic staff and relatives, typically children or grandchildren, turned out to be significant helpers. Often, the help consisted of downloading the app and navigating to the questionnaire, as illustrated by I17 *“We did not spend a lot of time on it, he (son of I17) downloaded the app and then I completed it”* (male, 75 years, G1). On the other hand, participants expressed navigation issues regarding to the questionnaire as illustrated by I12: “*I simply did not know where to find it [the questionnaire] in the app*” (female, 52 years, G2). In some cases, participants needed support throughout the entire process of completion due to technically inabilities. Typically, clinic staff helped during the startup phase and were available if challenges occurred; this level of help was stated as adequate by those who were not technically challenged, as illustrated by I23: “S*he [clinic staff] was available, I could just ask for help if needed, but it was not a problem [to complete PROMs]”* (female, 68 years, G1)*.* On the other hand, relatives typically sat next to the participant during the whole process of completion. In a few cases, participants expressed that helpers read out loud all questions and answers. For some participants, completion was only possible with the aid of the significant helpers, as illustrated by I6: *“Then I came to the nurse*, *and she helped with it [completion of PROMs]. Actually, I don’t think we could have filled it out at home”* (male, 79 years, G1)*.*

The time and location for completion of PROMs was flexible and there was a variation among participants as to whether they chose to complete PROMs at the outpatient clinic or at home. Eight participants in G1 completed PROMs at the outpatient clinic. Of those, two expressed they regretted completing PROMs at the outpatient clinic on the clinic staff’s advice, as they felt completion had to be done in a hurry. They would have preferred to complete PROMs in quiet surroundings at home. The remaining expressed satisfaction about flexibility in completion. Some completed PROMs during waiting times, which they preferred instead of postponing it until they were at home*.* The choice of whether to complete at home or in the outpatient clinic depended on the clinic staff’s advice, and probably also on whether the participant expressed capability in completing it independently, as illustrated by I26: *“I chose to complete it [the PROMs] at home (…) I could easily handle it at home” (male, 68 years, G1).*

### Suggestions for use of PROMs

Almost all participants in G3 expressed that they declined to complete PROMs due to insecurity about electronics. Some wished to complete PROMs in paper form instead of electronically. Participants also suggested that data could be gathered through conversation with health professionals instead. Participants from G2 proposed to have a link to the questionnaire sent to their mailbox to support user-friendliness, as participants with limited electronically capabilities would have easier access to the questionnaire through a known user interface. The only improvement in the questionnaire that participants in G1 proposed was the possibility of writing comments in text boxes.

## Discussion of results

Most of the participants had a sparse understanding of the purpose of completing PROMs. Recent studies reported that some participants did not know what their reported data were used for [33–35]. According to Primdahl et al., participants’ motivation to answer PROMs was affected by lack of knowledge about the purpose of collecting PROMs [36]. It is likely that a similar connection as found by Primdahl et al. could have occurred among participants in G2 and G3 [36], and the motivation for completion increased if they knew that PROMs were used as follow-up tools with relevance for each single patient. Nevertheless, it did not seem like the motivation among participants in G1 was influenced by their sparse knowledge, as everyone completed PROMs. A higher degree of motivation must affect the response rate positively, which is important to ensure, due to usage of PROMs as a follow-up tool and to apply PROMs in research to clarify the outcomes representing the whole patient population, not only the participants who manage to complete PROMs.

The degree in which a person perceives stimuli as cognitively understandable is incorporated in the theory *sense of coherence* developed by Antonovsky [37]. The theory consists of three elements: comprehensibility, manageability, and meaningfulness [37]. In this context, comprehensibility is relevant, as it appears from the interviews that some of the participants might have perceived the information in the outpatient clinic as structured, coherent, and clear, while others found the information disorderly, random, and as inexplicable “noise”. This difference is rooted in whether the participant find the information as having a high or low degree of comprehensibility, which affects the participant’s sense of coherence in a positive or negative way [37]. Robust communication strategies that support the participants’ ability to comprehend the information, by clarifying the purpose of PROMs’ collection, must, in future implementation of PROMs, enrich the patient experience and result in a higher response rate. The motivation to complete PROMs was reduced among participants in G3 due to their electronic inabilities. A possible contributing cause of rejection of PROMs’ completion might be due to the participants age, as all participants in G3 is more than 75 years old and they perhaps are not used to manage electronics. This consideration is supported by a recent study, which identified an age difference of two years between responders (69 years) and non-responders (71 years) [38]. In future research, it would be highly relevant to assess the challenges specifically experienced by the elderly participants completing PROMs, to focus an effort to remedy challenges in completing PROMs.

Participants pointed out that pain was a challenging subject to report in PROMs, due to pain variation during the day, the impact of painkillers, and distinguishing between osteoarthritis and other comorbidities. In accordance with other studies, patients were challenged when reporting on pain in PROMs [39–41]. The challenges occur due to lack of coverage of pain medication, the recurring following activity, and distinguishing the hip pain from other comorbidities causing pain when only answering the Oxford Hip Score (OHS) [39, 40]. The authors argue that the Oxford Hip Score (OHS) is deficient when patients report on pain [39]. In the current study, the OHS was supplemented by a question regarding the frequency of use of painkillers. Nevertheless, the question does not cover the participants’ experience of pain when taking painkillers, and in which degree participants’ pain rating is effected by the intake of painkillers. For future implementation of PROMs, we first recommend adding a clear introductory text with questions relating to pain, to accommodate participants’ confusion about considering painkillers or not when answering. Secondly, questions should be added concerning comorbidities and the effect on pain rating that the intake of painkillers has, to fully cover pain experienced by participants.

The ease of completing PROMs varied among participants. Some expressed that helpers were of great importance during completion. An English study also identified that participants completed OHS with varying levels of ease [41]. The element of manageability in the theory *sense of coherence* [37] can shed light upon this finding as it refers to a person’s balance between their resources and the requirements placed upon them. When the participants did not have the required electronic capabilities to complete PROMs, helpers compensated, whereby completion of PROMs became possible. Dialogue ensuring patients have the needed resources to complete PROMs is of importance when asking patients to complete PROMs.

Participants who felt challenged using electronic devices requested a possibility to complete PROMs in paper form. This is in accordance with findings in metastatic breast cancer patients, as some responded it was easy to use electronic data collection for PROMs, while others were more comfortable with paper [35]*.* Taking PROMs to the next level in clinical practice, findings by Marshall et al. integrate PROMs as a patient decision aid, in terms of setting realistic expectations for patients and to promote shared decision-making [12]. They concluded that decision quality improved for patients who used the decision aid [12].

### Study strengths and limitations

The exploratory approach of conducting qualitative individual interviews was useful, as the individual interview allowed the participants in the study to share their experiences openly without worrying about other participants’ experiences or their own electronic capabilities, which could be a challenge when performing focus group interviews instead. As TKA/THA, being in many ways an elective procedure with directly observable and felt outcomes, it is obvious to use PROMs. The main strength in this study is that, to our knowledge, it is one of few studies to examine patients’ experiences with the use of PROMs during an elective surgery course in patients undergoing TKA/THA. This study contributes with knowledge of challenges and barriers for completing PROMs in a Danish orthopedic setting and provides knowledge of how the setting, electronics and content of PROMs is received by the participants. This study could help qualify and tailor the PROM questionnaire to be more user-friendly and easy accessible. Another strength of the study is that we succeeded in obtaining a variated study population in terms of age, sex, type of operation, and degree of PROMs completion. However, it could have been relevant to add a cognitive screening to our inclusion criteria to ensure valid information, as it became clear during interviews that some patients were unsure what the PROMs was, and which information they received in the outpatient clinic was of relevance during interviews. A limitation could be that participants might not have been able to process the information giving pre-operatively. As the time from being informed in the outpatient clinic until interviews were conducted varied, this could introduce recall bias. To reduce recall bias, we performed interviews as quick as possibly after participants had received information in the outpatient clinic. The authors were aware of the possibility of recall bias that could occur in term of introduction to PROMs, when including participants answering PROMs up to three months post-surgery, considering the time gone since they received information about PROMs. Nevertheless, investigating whether participants were able to remember the content of PROMs and to complete PROMs singlehandedly was of importance, as PROMs are developed as a tool used at follow-up in the clinic.

## Conclusion

This study showed that the majority of the 33 included participants scheduled for TKA/THA was not fully aware of the purpose of completing PROMs. Motivation to complete PROMs occurred from a desire to help others; either scientist, clinic staff, or future patients. Demotivation was affected by electronic inabilities. In terms of completing PROMs, the participants in the study expressed a varied ease of use, with some perceived technical challenges. The participants expressed satisfaction with the flexibility of completing PROMs in outpatient clinic or at home; nevertheless, some did not manage completion on their own. Helpers were of great importance for completion, especially for participants with limited electronic capabilities.

## Data Availability

The datasets analyzed are available from the corresponding author on reasonable request.

## Abbreviations

APP: Application
OHS: Oxford hip score
PROMs: Patient-reported outcome measures
THA: Total hip arthroplasty
TKA: Total knee arthroplasty
